# From flower to fruit: fruit growth and development in olive (*Olea europaea* L.)—a review

**DOI:** 10.3389/fpls.2023.1276178

**Published:** 2023-11-13

**Authors:** Adolfo Rosati, Enrico Maria Lodolini, Franco Famiani

**Affiliations:** ^1^ Consiglio per la ricerca in agricoltura e l’analisi dell’economia agraria (CREA), Centro di ricerca Olivicoltura, Frutticoltura e Agrumicoltura, Spoleto, Italy; ^2^ Dipartimento di Scienze Agrarie, Alimentari e Ambientali, Università Politecnica delle Marche, Ancona, Italy; ^3^ Dipartimento di Scienze Agrarie, Alimentari e Ambientali, Università degli Studi di Perugia, Perugia, Italy

**Keywords:** cell number, fruit size, blooming, fruit set, ovary, pistil abortion, sink strength, yield components

## Abstract

The olive (*Olea europaea* L.) is the most cultivated tree crop in the Mediterranean and among the most cultivated tree crops worldwide. Olive yield is obtained by the product of fruit number and fruit size; therefore, understanding fruit development, in terms of both number and size, is commercially and scientifically relevant. This article reviews the literature on fruit development, from the flower to the mature fruit, considering factors that affect both fruit size and number. The review focuses on olive but includes literature on other species when relevant. The review brings the different factors affecting different phases of fruit development, addressed separately in the literature, under a single frame of interpretation. It is concluded that the different mechanisms regulating the different phases of fruit development, from pistil abortion to fruit set and fruit size, can be considered as different aspects of the same overall strategy, that is, adjusting fruit load to the available resources while striving to achieve the genetically determined fruit size target and the male and female fitness targets.

## 1 Introduction

The olive (*Olea europaea* L.) tree is the most cultivated tree crop in the Mediterranean, and one of the most cultivated trees worldwide, covering approximately 10 M hectares in 2021 (FAO, 2023). Olive yield is given by the number of fruits produced and their size; therefore, both fruit number and size are relevant aspects, both commercially and scientifically. In olive, the fruit develops directly from the carpel in the flower (King, 1938), and thus, fruit size is obtained through ovary development, before, during, and after flowering. Fruit number is also related to flower development, via flower number and fruit set. Therefore, this review considers fruit development from bloom to ripe fruit, including and integrating both fruit size and fruit number.

## 2 Factors affecting fruit size

Fruit size results from the interaction of environmental factors and the fruit growth potential, which is genetically determined. The olive fruit is a drupe: a fruit in which the mesocarp and endocarp tissues represent the major proportion of the fruit (King, 1938). In olive, fruit size is genetically controlled (Padula et al., 2008), differing many folds among different cultivars (Barranco, 1999). In general, fruit size differences within and among cultivars can be explained by different cell numbers, cell sizes, and/or intercellular spaces (Bertin et al., 2002; Corelli-Grappadelli and Lakso, 2004). In olive, genotype differences in fruit size are mostly due to cell number (Rapoport et al., 2004; Rosati et al., 2011a), as in many other species, such as tomato (Bohner and Bangerth, 1988), peach (Scorzal et al., 1991; Quilot and Génard, 2008), barley (Tuberosa et al., 1992), strawberry (Cheng and Breen, 1992), avocado (Cowan et al., 1997), melon (Higashi et al., 1999), banana (Jullien et al., 2001), tomato (Bertin et al., 2003), and persimmon (Hamada et al., 2008). Nonetheless, from the ovary to the fruit, the fruit grows mostly via increased cell size, rather than via increased cell number, as found in olive (Rapoport et al., 2004; Hammami et al., 2011) and, more in general, in fleshy fruits (Coombe, 1976). Accordingly, when considering the whole volume of the ovary/fruit, cell number increases approximately 8–40 times in the mature olive, compared with the ovary, while cell size increases approximately 100–300 times (Rallo and Rapoport, 2001; Rapoport et al., 2004; Rosati et al., 2009; Hammami et al., 2011; Rosati et al., 2012). This is related to the fact that cell division stops early during fruit formation (approximately 6 weeks after bloom), while cell expansion continues longer, especially in the mesocarp (Rallo and Rapoport, 2001). At times, however, fruit size differences are also related, at least in part, to cell size differences, as found in peach (Yamaguchi et al., 2002) and in apple, at least between wild relatives and cultivated varieties (Harada et al., 2005). A negative correlation between cell size and cell number is often found in mature fruits, suggesting resource competition among cells (Tsukaya, 2006). In olive, this correlation was weak across cultivars with different fruit sizes, although a boundary line analysis revealed that the maximal cell size achievable can be limited by high cell numbers, even though the size achieved is usually smaller and thus not limited by cell number (Rosati et al., 2011a).

Within a genotype, fruit size is affected by environmental (or exogenous) conditions affecting either cell size, cell number, or both (Denne, 1960; Bergh, 1985; Costagli et al., 2003; Gucci et al., 2009). These include nitrogen availability (Fernandez-Escobar et al., 2008), water availability (Costagli et al., 2003; Gucci et al., 2009), and solar radiation (Trentacoste et al., 2017). Irrigation, for instance, has been found to increase fruit size in olive mostly via increasing cell size (Costagli et al., 2003; Gucci et al., 2009). However, it has been hypothesized that water stress decreases fruit size by reducing cell number, when applied during early fruit development, and by reducing cell size, when applied later (Beede and Goldhamer, 1994; Orgaz and Fereres, 1999). In apple, heavy thinning before bloom increases fruit size by increasing both cell number and their size (Denne, 1960), although in the year following an abundant crop, fruit size is reduced via reduced cell number, and the reduction already occurs in the flower primordia in the previous autumn (Bergh, 1985). In olive fruits with multiple seeds, fruit size depends also on seed number, as found in Hojiblanca (Cuevas and Oller, 2000). Altogether, these results suggest that when resources per flower/fruit are increased (or decreased) at a time when the fruit tissues are still cellularizing (i.e., before, during, and up to sometime after bloom), then fruit size will be increased via increased cell number. When resources per fruit are varied after the completion of cellularization (as with irrigation treatments imposed in the summer after cellularization is complete), then fruit size differences can be achieved only by variations in cell size. When resources are varied during a period extending both before and after cellularization, both cell number and cell size will likely be affected. Further analysis of environmental factors affecting fruit size is not considered in this review, which mainly focuses on endogenous factors, particularly on genetic differences and their mechanisms.

### 2.1 Contribution of mesocarp and endocarp to ovary and fruit size

Despite evidence that both endocarp and mesocarp explain the differences in fruit size, in many fruit species (McGarry et al., 2001; Yamaguchi et al., 2004; Olmstead et al, 2007), including olive (Hammami et al., 2011; Rosati et al., 2012), on most species there is no information on whether this occurs also in the ovaries. In olive, both ovary endocarp and mesocarp volumes are strongly correlated with ovary volume, both across and within cultivars differing in fruit size (Rosati et al., 2012), implying a strong proportionality in tissue size. Even the size of locules is closely correlated with both tissues and with the ovary as a whole (Rosati et al., 2012). There is proportionality also between the ovary and the other flower parts, both within the same tree (Cuevas and Polito, 2004) and among cultivars (Rosati et al., 2009).

### 2.2 Relations between fruit and ovary tissues

In olive, mature fruit size is correlated to ovary size at bloom across cultivars differing in fruit size (Rosati et al., 2009), as occurs in other species (Lai et al., 1990; Lawes et al., 1990; Scorzal et al., 1991; Cruz-Castillo et al., 1991; Cheng and Breen, 1992; Nesbitt and Tanksley, 2001; Handley and Dill, 2003). In olive, this correlation holds also for both the endocarp and mesocarp although the relationships differ (i.e., different slopes), the relative increase being greater for the mesocarp (Rosati et al., 2012). This relative increase, from bloom to ripe fruit, represents the relative growth (RG) of the tissue: the final tissue volume (or mass) in the fruit per unit of initial volume (or mass) in the ovary. In terms of volume, RG varies across cultivars, from approximately 400 to 1,000 mm^3^ mm^−3^ for the endocarp and from approximately 1,700 to 7,000 mm^3^ mm^−3^ for the mesocarp (Rosati et al., 2012). RG for the whole fruit has intermediate values (Rapoport et al., 2004: Rosati et al., 2009). The greater RG for the mesocarp is related to its longer growth time compared with the endocarp (Hammami et al., 2011; Rapoport and Moreno-Alías, 2017).

### 2.3 Tissue relative growth vs. initial cell size in the ovary

Across tissues (mesocarp and endocarp) and cultivars, RG is negatively and exponentially correlated with the initial cell size of that tissue and cultivar in the ovary (Rosati et al., 2012). This is the case also when including data from a tetraploid cultivar with much bigger cells in the mesocarp and endocarp tissues in the ovary (Rosati et al., 2020). Therefore, the size of the cells in the ovary tissues appears to indicate how far that tissue is in its growth process: bigger cells indicate that less growth remains to occur. In fact, from bloom to harvest, tissue size increases continuously (Sinnott, 1942; Jullien et al., 2001; Bertin, 2005; Harada et al., 2005; Hammami et al., 2011), and daughter cells, before dividing again, grow to a bigger size than did their mother cell (Sinnott, 1942). This implies that cell size can be used as an indication of the stage of tissue development.

The fact that a single regression between RG and initial cell size fits both tissues suggests that both mesocarp and endocarp have a similar relationship between cell size and timing of tissue development. In other words, the larger cells of the endocarp than of the mesocarp at bloom suggest that the endocarp is at a more advanced growth stage, with more differentiated cells, and thus, its remaining growth from bloom to cessation of tissue growth (RG) is less. As already mentioned, the endocarp ceases growth earlier than the mesocarp and has a lower RG. This hypothesis is plausible since in the endocarp vital functions (e.g., fertilization) are required soon after anthesis, and thus, its cells need to be more differentiated. The mesocarp cells, instead, do not perform particular functions at anthesis and only need to prepare for their potential growth, thus not needing to achieve advanced differentiation in this period. However, even within the mesocarp, by 4 weeks after anthesis and up to fruit maturity, there is a gradient in cell size, with outside cells remaining smaller (and rounder) than inside ones (Rallo and Rapoport, 2001), suggesting that the centrifugal gradient of cell differentiations is not only between tissues (the endocarp having bigger and more differentiated cells than the mesocarp) but also within tissues.

### 2.4 Fruit tissue size dependence on ovary tissue cell number

In the previous sections, it has been described how, in olive, cultivar differences in fruit (and fruit tissue) size are related to ovary (and ovary tissue) size. In turn, differences in ovary and fruit size are related to cell number, for both the endocarp and the mesocarp (Rapoport et al., 2004; Rosati et al., 2011a). Therefore, for both these fruit portions, tissue size in the fruit correlates also with tissue cell number in the ovary (Rosati et al., 2012). This occurs also in other species such as apple (Denne, 1960; Harada et al., 2005), peach (Scorzal et al., 1991), kiwi (Cruz-Castillo et al., 1991), strawberry (Cheng and Breen, 1992), tomato (Bohner and Bangerth, 1988; Nesbitt and Tanksley, 2001), saskatoon (McGarry et al., 2001), and persimmon (Hamada et al., 2008), where fruit size and cell number in some fruit tissue correlate with the cell number of the corresponding tissue in the ovary. In most fruit species, this correlation has been studied for one tissue, but not for both the endocarp and the mesocarp. In olive, both the endocarp and the mesocarp provide important contributions to the fruit, despite differing in their growth patterns and timing (Hammami et al., 2011; Rapoport and Moreno-Alías, 2017), and despite showing different characteristics in the ovary, the endocarp having approximately half the number of cells of approximately twice the size than the mesocarp (Rosati et al., 2011a). Notwithstanding these differences, fruit tissue size strongly correlates to ovary tissue cell number, with a similar quantitative relationship for both tissues (Rosati et al., 2012; Rosati et al., 2020), which implies that the endocarp and mesocarp produce a similar mass in the fruit, for each cell in the ovary, despite having different cell sizes and numbers at anthesis. This could derive from a similar cell division rate for the two tissues, while the smaller cells of the mesocarp allow for a more extended period of cell expansion (explaining the longer growth of the mesocarp). This would lead to both tissues having a similar cell size in the ripe fruit. A similar cell division rate, combined with a similar final cell size, would lead to a similar final number of cells in the fruit, of similar size, for every ovary cell, notwithstanding initial cell size differences in the ovary. Accordingly, in young cucurbit ovaries, cell division rate is very similar for all tissues, despite different cell sizes and differentiation level, the inner tissues having bogger cells than the outer tissues (Sinnott, 1942), as also found in olive. Unfortunately, no studies report the cell size in the olive endocarp, probably because of the eventual lignification of this tissue, which makes measurements difficult.

### 2.5 Cell number rather than tissue mass determines sink strength

As mentioned in the previous sections, in olive, fruit endocarp and mesocarp size at harvest correlates with tissue cell number in the ovary, with a single quantitative relationship across tissues and genotypes (Rosati et al., 2012; Rosati et al., 2020), suggesting that the growth potential and sink strength of both tissues are functions of their cell number. In some species, like melon (Higashi et al., 1999), pear (Zhang et al., 2006), and cherry (Olmstead et al, 2007), genetic differences in fruit size and cell number arise from different extents of cell division after antheses, instead of before. Nonetheless, fruit size still correlates with fruit cell number. This suggests that cell number is a valid indicator of sink size in fruits (Ho, 1988, Ho, 1992; Bertin, 2005; Génard et al., 2007) although cell number is not the only factor affecting sink strength (Gillaspy et al., 1993; Marcelis et al, 1998). This agrees also with the suggestion that cell division before bloom determines potential fruit size (Ho, 1996), even if an important amount of cell division continues after bloom (Scorzal et al., 1991), as is the case in olive (Hammami et al., 2011). In fact, ovary cell number before anthesis is considered a critical determinant of the sink strength of the developing fruit and is genetically controlled (Coombe, 1976). However, within tissues, cell size is similar across olive cultivars, in the ovary (Rosati et al., 2011a) as well as in the fruit (Rapoport et al., 2004), and thus, cell number correlates closely with tissue size. Therefore, the correlation between tissue cell number in the ovary and tissue size in the fruit results in correlations also between tissue size in the ovary and tissue size in the fruit although, in this case, not with a single quantitative relationship but with different regressions between tissues (Rosati et al., 2012). Thus, a causal relationship between tissue growth and tissue mass cannot be excluded.

Sink strength is the product of sink size and sink activity (Warren Wilson, 1972). This is often interpreted as the product of sink mass and relative growth rate (RGR) (Marcelis et al., 1998). However, RGR decreases exponentially during fruit development (Bangerth and Ho, 1984; Marcelis, 1992; Grossman and DeJong, 1995), and there is no mechanistic explanation for this decrease. An alternative approach is that of Ho, 1988; Ho, 1992 and Génard et al. (2007), who suggest that cell number represents sink size while the cell growth rate potential represents sink activity. The first approach (i.e., sink strength = fruit mass × RGR) is more practical since fruit mass is more easily measured than cell number. For this reason, it has been extensively used for fruit growth modeling (e.g., Grossman and DeJong, 1994). Such models, however, are not mechanistic but only phenomenological, and there is no evidence of a causal relationship between mass and sink size; on the contrary, there is evidence that there is no causal link (Marcelis, 1996). Starck and Ubysz (1974) reasoned that growth rate rather than organ size determines the partitioning of dry matter into an organ. In fact, in other models, sink size is represented by cell number and sink activity by cell growth rate (Génard et al., 2007; Fanwoua et al., 2013).

While both approaches work, empirically, it remains to be determined whether fruit (and tissue) growth is determined by tissue size or cell number. As discussed above, the fact that, at least in olive, tissue size in the fruit correlates with cell number in the ovary with a single relationship across tissues, while separate relationships occur between tissue size in the fruit and tissue size in the ovary, suggests that cell number rather than tissue mass drives tissue growth (Rosati et al., 2012). Further evidence for this stems from the observation that fruit growth is initially exponential, then virtually linear, both in olive and in other species, like apple, where this pattern has been defined as an expolinear growth model (Lakso et al., 1995). The exponential phase of the growth overlaps roughly with the cell division stage, while the linear growth occurs later when cell division ceases or slows down considerably. In fact, even differences in growth rate between different crop load treatments were related to differences in fruit cell number (Lakso et al., 1995), implying that the fruit growth rate per cell is constant. These observations suggest that fruit growth rate is proportional to cell number during the whole growth period, increasing while cell number increases, then remaining constant when cell number is constant. Further evidence that cell number (and not organ mass) drives fruit growth was obtained by using the diploid olive cultivar Leccino and its tetraploid genotype (Rosati et al., 2020). The tetraploid genotype was obtained by mutagenesis (Rugini et al., 2016) and has bigger ovaries, but the bigger size results from larger cells in approximately equal numbers (Caporali et al., 2014), unlike usually found across cultivars, where cell number explains different ovary sizes (Rosati et al., 2011a). The larger ovaries of the tetraploid grew at a similar absolute rate (thus lower RGR) and reached a similar final size as the diploid cultivar (instead of a proportionally greater size), breaking the rule that final tissue mass in the fruit correlates with initial tissue mass in the ovary (Rosati et al., 2020). However, the final tissue mass in the fruit remained correlated to initial cell number in the ovary, strongly supporting the hypothesis that it is the cell number and not the tissue mass that is causally linked to tissue growth.

### 2.6 Further considerations about the dependence of fruit growth on cell number

The hypothesis that fruit growth is causally related to cell number is supported by the fact that, in many species, such as tomato (Nesbitt and Tanksley, 2001) and many others (reviewed in Guo and Simmons, 2011), fruit size is affected by genes (such as fw2.2), controlling cell division before bloom. Similar genes have been found also in olive (Cirilli et al., 2012; Cirilli et al., 2013). These genes act generally across species and organs (Guo et al., 2010). Therefore, in olive, fruit growth appears to be controlled by genes regulating cell number in the ovary tissues before bloom. In fact, considering that all flower parts (petals and stamens) also vary in size with the ovary (Rosati et al., 2009), it seems that these genes act on all flower parts and tissues.

Genes regulating cell division before bloom might not control fruit size in other species where fruit size differences across cultivars result from different post-bloom duration of cell division, rather than from differences in ovary size and cell number at bloom, as in melon (Higashi et al., 1999), pear (Zhang et al., 2006), and cherry (Olmstead et al, 2007). In such species, fruit size might be controlled by genes that regulate the duration of cell division after anthesis. Similarly, fruit size might not correlate with ovary size in species where the fruit does not develop directly from the ovary but derives from secondary tissues that cellularize after bloom, as in cereals (Benincasa et al., 2017; Benincasa et al., 2022). The expression of genes regulating cell division and thus fruit size is still subject to the availability of resources, as shown in tomato (Baldet et al., 2006). Inadequate nutrition induces proportional sterility by increasing ovary abortion or even failure to flower (Marcucci, 1950).

## 3 Factors affecting flower and fruit number

Olive trees have abundant flower production but low fruit set: typically, only 1%–3% of flowers turn into a fruit (Hartmann, 1950). Fruit set depends on flower quality, including pistil abortion, and on factors occurring both before and after flowering. This review considers the effects of ovary size and cell number (and the consequent sink strength and competition ability) on pistil abortion and fruit set. Evolutionary reasons for abundant flowering and low fruit set in this species will also be considered.

### 3.1 Fruit size and pistil abortion

Pistil or ovary abortion is an expression indicating the presence of flowers with absent or only partly formed ovaries, thus ovaries that are unable to develop into fruits. Such flowers are called staminate flowers and have only the male organs fully developed and functional. Normal flowers, which are the majority in olive, have both female and male organs fully developed and functional and are called hermaphrodite flowers. In olive, pistil abortion varies largely with cultivar and year, but also among trees for the same cultivar and within a single tree, among branches and shoots, as well as among and within inflorescences (Morettini, 1939; Bottari, 1951; Badr and Hartmann, 1971; Fabbri et al., 2004; Martin and Sibbett, 2005). Pistil abortion appears to occur at an early time (30–40 days before anthesis) during flower development (Pirotta and De Pergola, 1913; Uriu, 1959; Cuevas et al., 1999; Reale et al., 2006).

In olive, pistil abortion results mostly from resource competition since resources are insufficient for all flowers to develop, given the abundant flowering. This competition starts early, affecting both ovary abortion and fruit set (Hartmann, 1950; Uriu, 1959; Cuevas et al., 1994; Perica et al., 2001; Levin and Lavee, 2005). Conditions that decrease available resources or increase competition among flowers and fruits usually increase pistil abortion and decrease fruit set. These conditions include N deficiency, water stress (Melis, 1923; Brooks, 1948; García Gálvez, 2005; Rapoport et al., 2012), heat stress (Benlloch-González et al., 2018), and insufficient photosynthetically active radiation (Bottari, 1951; Dimassi et al., 1999). They also include foliar diseases and low leaf-to-bud ratio (Petri, 1920; Morettini, 1951; Uriu, 1953; Uriu, 1959; Fernandez-Escobar et al., 2008), abundant flowering (Reale et al., 2006) or inflorescence position in the canopy that are unfavorable (Cuevas and Polito, 2004; Seifi et al., 2008), and adverse weather and high previous-year yield (Rallo et al., 1981; Rapoport and Rallo, 1991; Cuevas et al., 1994; Lavee, 1996). The fact that aborted flowers do not contain starch also suggests a link between trophic levels and ovary abortion (Reale et al., 2009). Additionally, when the distal half of the inflorescences are removed, ovary abortion decreases drastically in the remaining flowers (Seifi et al., 2008).

Ovary abortion is also known to vary across cultivars (Campbell, 1911; Morettini, 1939; Morettini, 1951; Magherini, 1971; Lavee, 1996; Lavee et al., 2002). In olive, Rosati et al. (2011b) suggested that even the genetic component of pistil abortion may be explained in terms of competition. In fact, larger-fruited cultivars often have greater rates of ovary abortion (Morettini, 1939; Magherini, 1971; Acebedo et al, 2000; Rosati et al., 2011b) but also larger ovaries and flowers (Rosati et al., 2009). This implies greater use of resources per flower and greater sink strength (i.e., greater competition ability) of individual flowers/ovaries, related to their greater cell number (Rosati et al., 2012; Rosati et al., 2020). As discussed above, greater competition ability leads to greater abortion.

### 3.2 Fruit set and fruit size

As already mentioned, in olive, the fruit set is low (Hartmann, 1950). It is tempting to assume that greater yields could be achieved by increasing fruit set. However, when the flower number is experimentally reduced, the fruit set increases, resulting in a similar fruit load (Suarez et al., 1984; Rallo and Fernandez-Escobar, 1985; Lavee et al., 1996; Lavee et al., 1999). This suggests a tendency of the tree to set a fixed mass of fruit, independent of the number of flowers. Previous authors refer to this fixed mass as the tree fruiting potential (Lavee et al., 1996), which reflects source availability. When the fruit set reaches such potential, the rest of the flowers will drop. Accordingly, poor nutrition decreases proportionally the plant’s fertility, completely blocking flower formation in extreme cases (Marcucci, 1950). Heat stress also decreases fruit set (Benlloch-González et al., 2018). In olive, competition for resources among flowers has been extensively reported (Suarez et al., 1984; Rapoport and Rallo, 1991; Cuevas et al., 1994; Cuevas et al., 1995; Lavee et al., 1999; Seifi et al., 2008; Rosati et al., 2010, Rosati et al. 2011b). Rugini and Pannelli (1993) found that fruit set increases when competition between flowers and shoots is relieved by mechanically or chemically slowing down shoot growth, further supporting the hypothesis that fruit set is limited by resource availability.

Rosati et al. (2010) found that large-fruited cultivars, which have larger ovaries and flowers, have a lower fruit set so that the total fruit mass is similar. This suggests that there is compensation across cultivars between fruit size and number. Similar results had been found by Rallo and Fernandez-Escobar (1985). Thus, genetic differences in fruit set also appear to be explainable in terms of competition for resources among ovaries/flowers/fruits of different sizes, that is, in large-fruited cultivars, larger ovaries have more cells and thus greater sink strength, therefore increasing competition and decreasing fruit set. This also explains why cultivars with small fruits, like Arbosana and Arbequina for instance, set several fruits per inflorescence, whereas cultivars with large fruits produce typically only one fruit, of bigger size, per inflorescence. It could be argued that small-fruited cultivars might produce small fruits as a result of higher fruit set and the consequent increase in source competition among fruits. This is unlikely the case because the fruit is already smaller at and before anthesis (i.e., smaller ovary), which is before the fruit set occurs. Additionally, when competition between fruits is reduced by thinning, fruit size increases only marginally (compared with possible differences among cultivars) and does not eliminate cultivar differences (Rosati et al., 2010), suggesting that these are genetically predetermined.

The final fruit size within a cultivar is still subject to the availability of resources, varying up to two-fold within cultivars. However, fruit size is much more variable among cultivars (Barranco, 1999), up to six-fold (Rosati et al., 2009). Therefore, regulating fruit load across cultivars with extremely different fruit sizes necessitates a greater compensation mechanism than the regulation of fruit size within cultivar: this mechanism seems to be the regulation of fruit set (i.e., higher fruit set in small-fruited cultivars).

### 3.3 Andromonoecy, redundant flowering, and fitness

As discussed above, the olive flowers redundantly, relative to its potential yield and fruit set, which is particularly low. We already discussed how pistil abortion increases at increasing resource competition since abortion allows resource-saving, matching the number of ovaries to the available resources (Primack and Lloyd, 1980; Bertin, 1982; Stephenson and Bertin, 1983). Plants that abort part of the ovaries in flowers that would otherwise be hermaphrodite, like the olive, are called andromonoecious. There are approximately 4,000 andromonoecious species (Yampolsky and Yampolsky, 1922; Richards, 1986; Miller and Diggle, 2003). In evolutionary terms, andromonoecy is considered an intermediate step toward dioecy, allowing to modulate resource allocation to the female function, in response to resource availability (Lloyd, 1980; Solomon, 1985; Sutherland, 1986; Diggle, 1994; Miller and Diggle, 2003). Andromonoecy allows to save resources that would be wasted in surplus ovaries, without affecting the plant’s number of flowers: this maintains the plant’s male function and fitness (Primack and Lloyd, 1980; Bertin, 1982; Stephenson and Bertin, 1983; Vallejo-Marín and Rausher, 2007). Pistil-aborted flowers have less mass (between 19% and 41% less) than hermaphrodite flowers in olive because ovaries and petals are smaller while stamens maintain their size and pollen production (Cuevas and Polito, 2004), implying resource-saving with andromonoecy.

**Figure 1:**
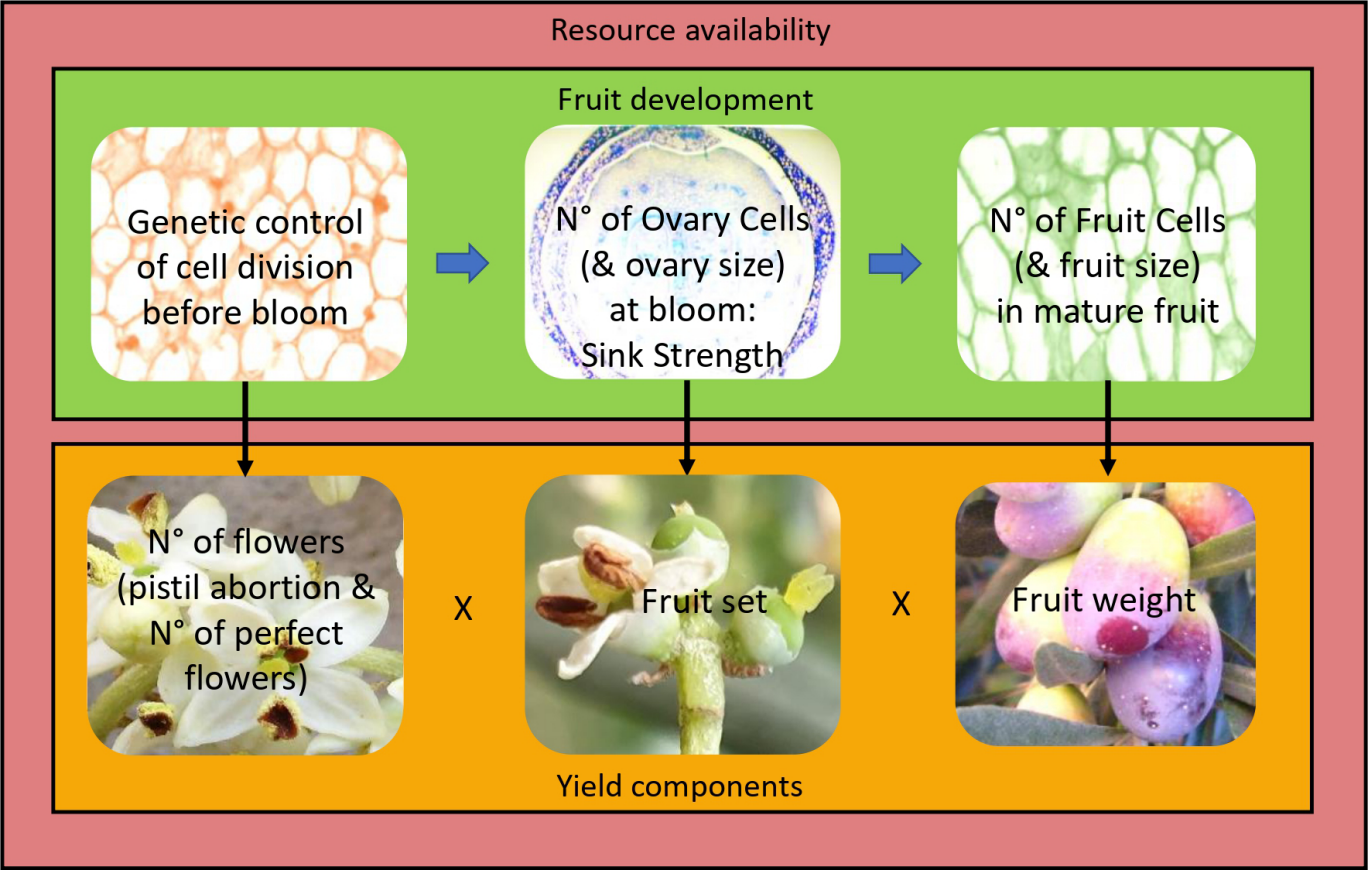
Proposed model of fruit development and yield formation. Cell division in the forming ovary (and other flower parts) is under genetic control. This determines cell number (and tissue size) and thus sink strength in the ovary at bloom. This in turn determines cell number in the fruit and potential fruit size. Yield is the product of the number of flowers (considering pistil abortion and thus number of perfect flowers) × fruit set × average fruit weight. All phases of fruit development and all yield components are modulated by resource availability (outside box), which is a function of the agronomic conditions (e.g., light interception, water and nutrient availability, biotic and abiotic stresses) and endogenous factors (e.g., alternate bearing, source–sink relationships, competition between vegetative growth and reproduction). Increasing resource availability will increase cell number and/or size and thus organ (flower/ovary/fruit) size at all stages of fruit development, as well as flower number, percent of perfect flowers, fruit set, and fruit weight. However, for a given resource budget, genetically larger flowers/ovaries/fruits, made up of more cells, will increase organ sink strength and competition. Thus, larger pre-anthesis flowers/ovaries will increase pistil abortion, and larger flowers/ovaries at bloom will decrease fruit set, in a compensatory manner between organ size and number, so that yield is virtually unaffected by organ size differences and mainly affected by the fruiting potential of the tree, as determined by resource availability. Arrows indicate how a fruit’s developmental stage affects the following stage (horizontal arrows) or a yield component (vertical arrows).

In olive, cultivars with large fruits have greater pistil abortion, but the number of flowers is similar to that of small-fruited cultivars (Rosati et al., 2010; Rosati et al., 2011b); thus, the male function (and fitness) remains probably unaffected. This explains why only the pistil is aborted and not the whole flower, despite the fact that aborting the whole flower would save much more resources, given that the flower is several times bigger than the ovary alone (Cuevas and Polito, 2004; Rosati et al., 2009). Aborting the whole flower would reduce the male fitness, while aborting only the ovary maintains the male function and thus the male fitness. **Figure 1** illustrates and summarizes the male and female fitness in the olive. Maintaining the male fitness also explains why the flower production is so excessive (i.e., low fruit set) compared with the fruiting potential of the tree: pollen production is less expensive than producing fruits and seeds while still assuring the male fitness. This makes it convenient to produce more (male) flowers than fruits. Morgan (1993) demonstrates that plants maximize their fitness when they produce many more flowers than fruits, and the optimal flower/fruit ratio is higher for andromonoecious and monoecious species compared with dioecious species. The optimal ratio increases further for anemophilous species, like the olive.

Therefore, the apparently redundant bloom in olive, while representing a waste of resources from an agronomical perspective, is useful to the tree in evolutionary terms, increasing the fitness of the plant. It might be desirable, therefore, to breed olive cultivars with reduced flowering (reduced male fitness), allowing the plant to save resources, which can then be invested in fruit set and development. In fact, the cost of flowering is significant in olive: inflorescence development consumes resources at a rate similar to that of fruit development (Famiani et al., 2019). Indeed, thinning up to 50% of inflorescences has resulted in increased overall fruit load per shoot (Lavee et al., 1999): this might be attributable to resource-saving for flowering. Preventing the formation of 95% of the inflorescences, rather than thinning 50% of them after their formation, could potentially save more resources and increase fruit load further.

## 4 Conclusions

From the reviewed literature, it may be concluded that, across olive cultivars of different potential fruit sizes, potential fruit growth is controlled by the ovary tissue characteristics at anthesis. Variations in ovary size, across and within cultivars, arise from parallel variations in the size of all tissues (i.e., endocarp and mesocarp). Understanding how sink strength and fruit (and tissue) growth are determined is of great importance, both scientifically and agronomically, and it is indispensable for developing mechanistic fruit growth models. From the literature quoted in this review, it may be concluded that, at least in olive, although fruit tissue size in the mature fruit generally correlates with ovary tissue size (with separate correlations for endocarp and mesocarp), tissue cell number is more likely to be the functional determinant of fruit and tissue growth. Therefore, fruit tissue size in olive appears to be controlled by genes regulating cell division before anthesis. The tissue RG, from anthesis to the fruit, is closely related to the size of the cells in the ovary, across both cultivars and tissues: this suggests that cell size in the ovary is indicative of tissue growth stage.

**Figure 2:**
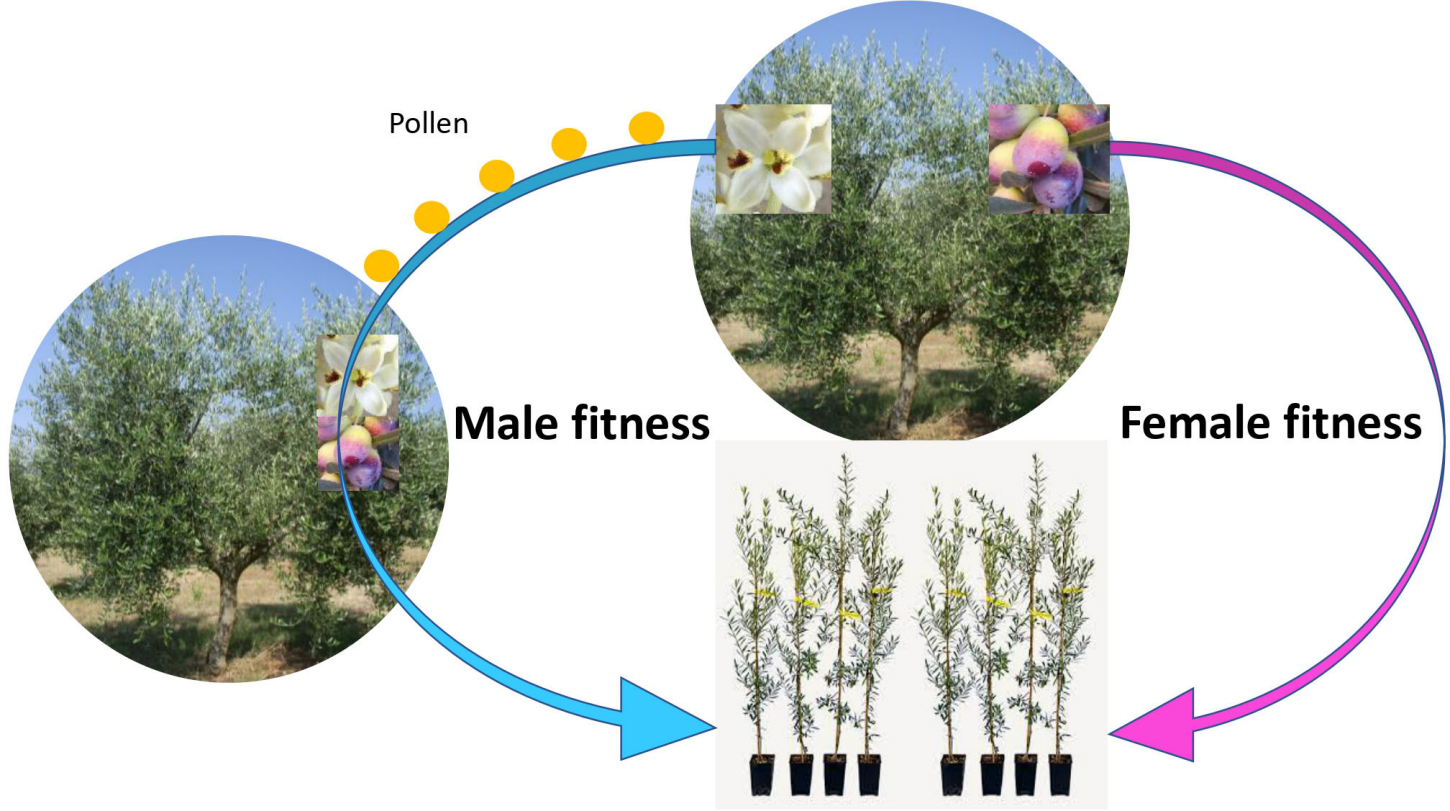
Schematic representation of male and female fitness. The tree invests in fruits to increase its female fitness (producing offsprings with 50% of the genome from the mother plant). However, producing pollen is just as effective at producing offsprings with 50% of the genome of the father plant, via pollinizing other trees. In fact, the biomass and resource investment per offspring might be smaller with the male fitness pattern. Redundant flowering (i.e., low fruit set) in olive, while being agronomically inefficient, is efficient in terms of fitness, thus in evolutionary terms.

Resource competition among developing flowers and fruits appears to play a continuous and fundamental role in adjusting fruit load to the available resources, during the whole fruit development. Pistil abortion first, then fruit set, fruit drop, and finally fruit size appear to be different and sequential mechanisms of this same strategy. Also, the genetic component (i.e., greater pistil abortion and lower fruit set in cultivars with larger ovary/flower/fruit size) can be explained with the competition hypothesis, based on the different sink strength and energy costs for the development of ovaries/flowers/fruits of different sizes. **Figure 2** illustrates and summarizes the interaction between resource availability, fruit development, and yield components. It can be concluded that, in the absence of dramatic events that might compromise fruit set and/or development (e.g., pollination problems, extreme drought, inadequate nutrition), the olive tree appears to regulate fruit load according to its yield potential, regardless of the number of flowers produced. Hence, correlations between the flowering amount (or the amount of pollen in the air) and yield (Moriondo et al., 2001; Fornaciari et al., 2002; Galán et al., 2004) do not entail a causal relationship. They just indicate that the phenomena are correlated: both flowering and yield levels reflect resource availability and the yield potential of the tree. The most effective way to increase yield, again in the absence of dramatic events that compromise fruit set and/or development, appears to be improving the tree’s ecophysiological status (i.e., improving nutrition and avoiding biotic and abiotic stresses), thus increasing its fruiting potential. The apparently redundant flowering in olive (i.e., low fruit set), although wasteful agronomically, serves the purpose of increasing the male fitness, which is an advantage in evolutionary terms. Selecting for reduced male fitness (i.e., reduced flowering) might allow to reallocate the saved resources to female fitness, possibly increasing yield somewhat.

## Author contributions

AR, FF: Conceptualization, Supervision, Visualization, Writing – original draft, Writing – review & editing. EML: Writing – review & editing.
