# Corrigendum: From flower to fruit: fruit growth and development in olive (*Olea europaea* L.)—a review

**DOI:** 10.3389/fpls.2025.1556708

**Published:** 2025-02-05

**Authors:** Adolfo Rosati, Enrico Maria Lodolini, Franco Famiani

**Affiliations:** ^1^ Consiglio per la ricerca in agricoltura e l’analisi dell’economia agraria (CREA), Centro di ricerca Olivicoltura, Frutticoltura e Agrumicoltura, Spoleto, Italy; ^2^ Dipartimento di Scienze Agrarie, Alimentari e Ambientali, Università Politecnica delle Marche, Ancona, Italy; ^3^ Dipartimento di Scienze Agrarie, Alimentari e Ambientali, Università degli Studi di Perugia, Perugia, Italy

**Keywords:** cell number, fruit size, blooming, fruit set, ovary, pistil abortion, sink strength, yield components

In the published article, there was an error in the legend for (**Figure 1** and **Figure 2**) as published. The Figure captions were inverted. The corrected legends appear below.

**Figure 1:**
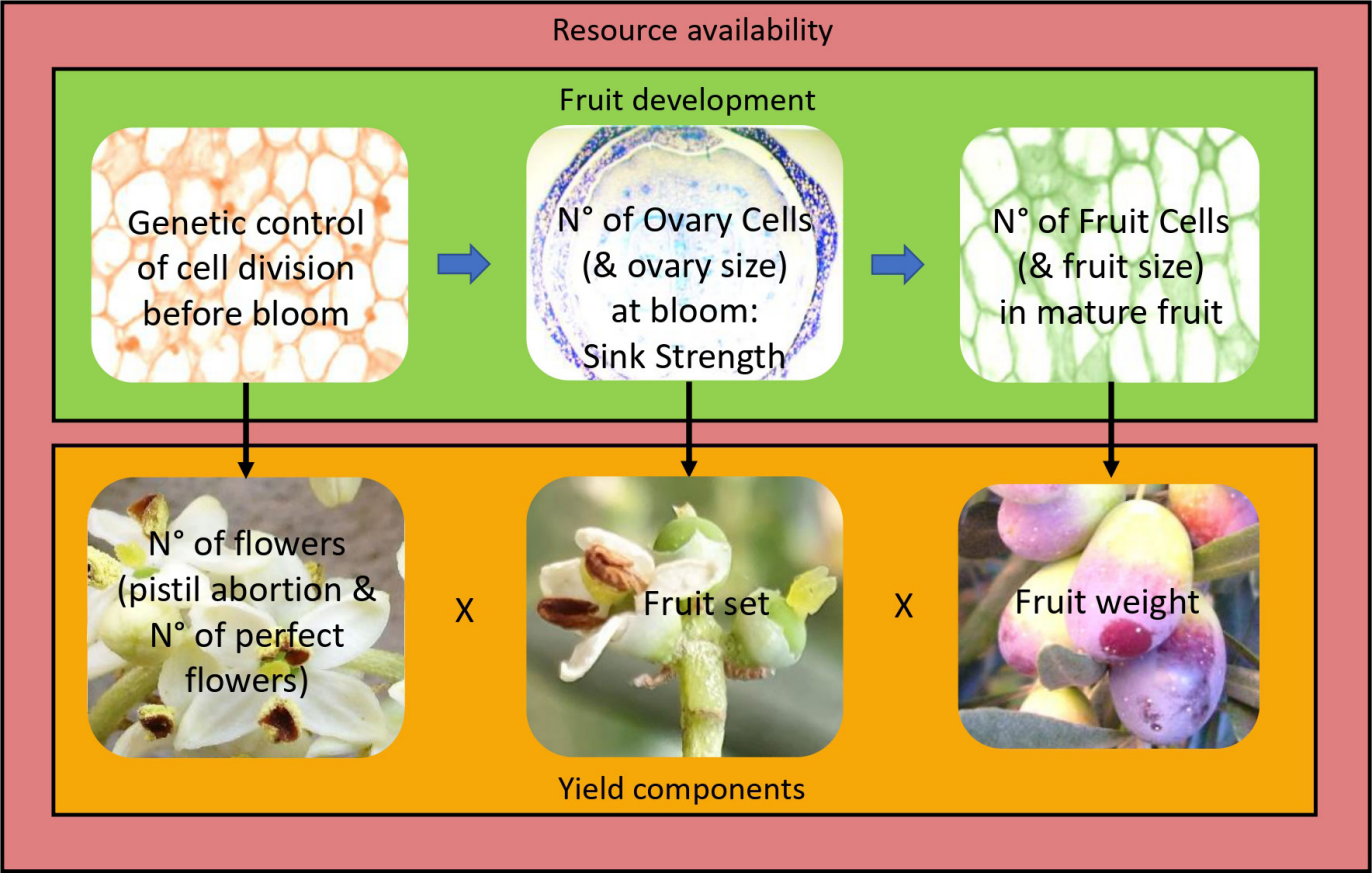
Proposed model of fruit development and yield formation. Cell division in the forming ovary (and other flower parts) is under genetic control. This determines cell number (and tissue size) and thus sink strength in the ovary at bloom. This in turn determines cell number in the fruit and potential fruit size. Yield is the product of the number of flowers (considering pistil abortion and thus number of perfect flowers) × fruit set × average fruit weight. All phases of fruit development and all yield components are modulated by resource availability (outside box), which is a function of the agronomic conditions (e.g., light interception, water and nutrient availability, biotic and abiotic stresses) and endogenous factors (e.g., alternate bearing, source–sink relationships, competition between vegetative growth and reproduction). Increasing resource availability will increase cell number and/or size and thus organ (flower/ovary/fruit) size at all stages of fruit development, as well as flower number, percent of perfect flowers, fruit set, and fruit weight. However, for a given resource budget, genetically larger flowers/ovaries/fruits, made up of more cells, will increase organ sink strength and competition. Thus, larger pre-anthesis flowers/ovaries will increase pistil abortion, and larger flowers/ovaries at bloom will decrease fruit set, in a compensatory manner between organ size and number, so that yield is virtually unaffected by organ size differences and mainly affected by the fruiting potential of the tree, as determined by resource availability. Arrows indicate how a fruit’s developmental stage affects the following stage (horizontal arrows) or a yield component (vertical arrows).

**Figure 2:**
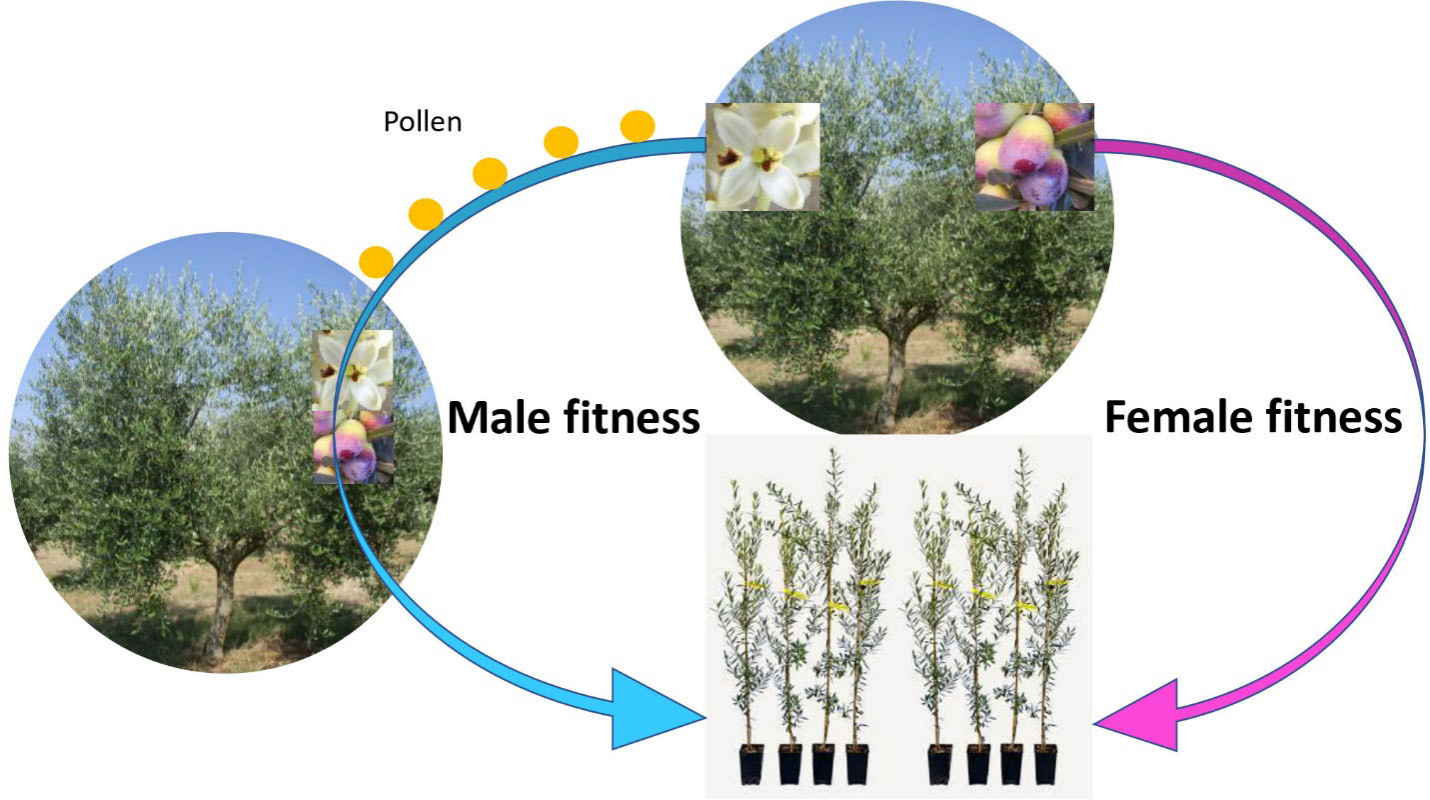
Schematic representation of male and female fitness. The tree invests in fruits to increase its female fitness (producing offsprings with 50% of the genome from the mother plant). However, producing pollen is just as effective at producing offsprings with 50% of the genome of the father plant, via pollinizing other trees. In fact, the biomass and resource investment per offspring might be smaller with the male fitness pattern. Redundant flowering (i.e., low fruit set) in olive, while being agronomically inefficient, is efficient in terms of fitness, thus in evolutionary terms.

The authors apologize for this error and state that this does not change the scientific conclusions of the article in any way. The original article has been updated.

